# Tetracycline does not directly inhibit the function of bacterial elongation factor Tu

**DOI:** 10.1371/journal.pone.0178523

**Published:** 2017-05-26

**Authors:** Katherine E. Gzyl, Hans-Joachim Wieden

**Affiliations:** Alberta RNA Research and Training Institute, Department of Chemistry and Biochemistry, University of Lethbridge, Lethbridge, Alberta, Canada; University of British Columbia, CANADA

## Abstract

Understanding the molecular mechanism of antibiotics that are currently in use is important for the development of new antimicrobials. The tetracyclines, discovered in the 1940s, are a well-established class of antibiotics that still have a role in treating microbial infections in humans. It is generally accepted that the main target of their action is the ribosome. The estimated affinity for tetracycline binding to the ribosome is relatively low compared to the actual potency of the drug *in vivo*. Therefore, additional inhibitory effects of tetracycline on the translation machinery have been discussed. Structural evidence suggests that tetracycline inhibits the function of the essential bacterial GTPase Elongation Factor (EF)-Tu through interaction with the bound nucleotide. Based on this, tetracycline has been predicted to impede the nucleotide-binding properties of EF-Tu. However, detailed kinetic studies addressing the effect of tetracycline on nucleotide binding have been prevented by the fluorescence properties of the antibiotic. Here, we report a fluorescence-based kinetic assay that minimizes the effect of tetracycline autofluorescence, enabling the detailed kinetic analysis of the nucleotide-binding properties of *Escherichia coli* EF-Tu. Furthermore, using physiologically relevant conditions, we demonstrate that tetracycline does not affect EF-Tu’s intrinsic or ribosome-stimulated GTPase activity, nor the stability of the EF-Tu•GTP•Phe-tRNA^Phe^ complex. We therefore provide clear evidence that tetracycline does not directly impede the function of EF-Tu.

## Introduction

Developing new antibiotics is a global priority as antibiotic-resistant bacteria are becoming more prevalent in common infections worldwide [1, 2]. There has been great investment in developing new antibiotics from chemical libraries, however, this approach has not been overly successful [3, 4]. The most promising route to developing new antibiotics to date has been through the modification of already known, naturally produced antibiotics [3]. However, resistance to these antibiotics usually occurs quickly because the respective resistance mechanisms are already present [4]. An alternative approach would implement known antibiotic molecular mechanisms while screening chemical libraries and rationally designing new small molecule inhibitors [3, 5]. However, from thousands of developed antibiotics, the molecular mechanism is only known for a few [3, 6]. Furthermore, little is known about the secondary and non-specific targets of these antibiotics. One of these antibiotics is tetracycline.

Tetracycline is a broad-spectrum antibiotic used in human and animal health with activity against a wide range of pathogens [7–10]. While tetracycline use has declined due to increasing antibiotic resistance, many tetracycline derivatives have been developed based on the core molecular structure of tetracycline. Newly developed tetracycline derivatives can bypass current resistance mechanisms [7, 11–14]. All tetracyclines, except for atypical tetracyclines that target the bacterial cytoplasmic membrane, bind to the 30S ribosomal subunit and sterically block aminoacyl (aa)-tRNA from being accommodated into the A site of the ribosome [7, 15]. The primary tetracycline-binding pocket is formed by the irregular minor groove of helix 34 and the stem loop of helix 31 in the 16S rRNA [13, 16, 17]. Tetracycline’s polar edge interacts with the sugar phosphate backbone of helix 34 and a magnesium ion, which coordinates indirect interactions with other nucleotides. A second magnesium ion coordinates interactions between tetracycline and helix 31. The hydrophobic face of tetracycline makes stacking interactions with bases of helix 34 [13]. These unspecific interactions and the chelating properties of tetracycline are the reason why tetracycline binding can also be observed for a number of secondary sites. The discrepancy between the minimal inhibitory concentration (MIC) and half maximal inhibitory concentration (IC_50_), as well as the diverse resistance mechanisms for tetracycline, support the functional relevance of tetracycline binding to these secondary binding sites [13, 18].

**Fig 1:**
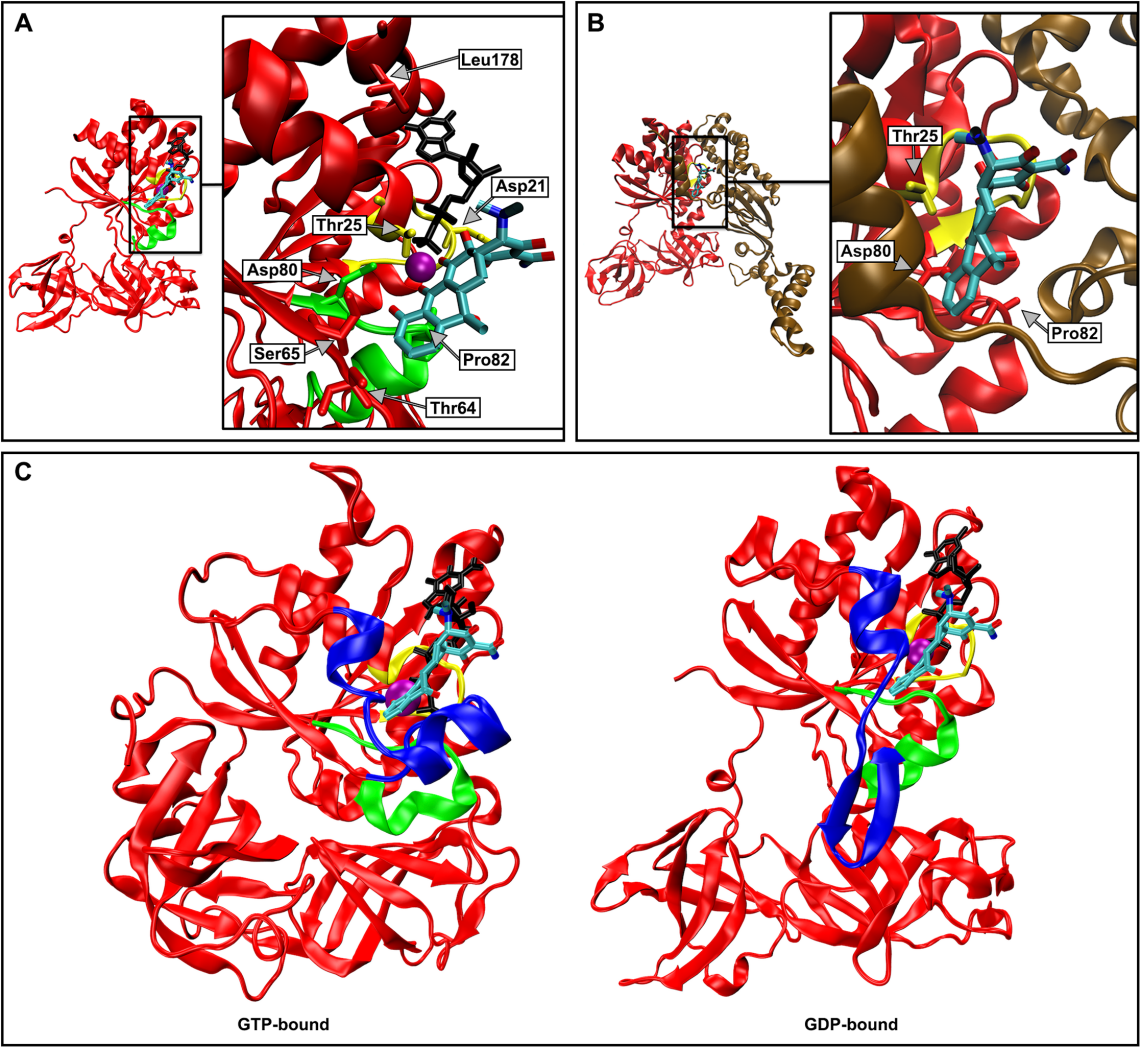
Structural comparison of tetracycline bound to different functional states of EF-Tu. Tetracycline is colored by atom, GDP and GTP are in black, and the magnesium ion is purple. In panel (A) the tetracycline-binding pocket is illustrated with the GTPase domain of EF-Tu (red) bound to GDP and tetracycline. Switch II is highlighted in green and the P-loop in yellow. Tetracycline is coordinated by the conserved magnesium ion, interacts with Thr25 (*E*. *coli* numbering), Asp80, and Pro82, and is in close proximity to Asp21, Thr64, Ser65, and Leu178. The X-ray structure of the EF-Tu•GDP•tetracycline complex (PDB ID 2HCJ) [21] was used to generate the cartoon illustration. Panel (B) shows that tetracycline binding is compatible with EF-Ts binding to EF-Tu•GDP. The interaction between tetracycline-bound EF-Tu•GDP (red) and EF-Ts (brown) is modeled, with the phosphate-binding loop highlighted in yellow. Domain I of EF-Tu in the EF-Tu•EF-Ts (PDB ID 1EFU) [33] crystal structure was superimposed onto domain I of EF-Tu•GDP•tetracycline (PDB ID 2HCJ) [21]. Panel (C) shows that tetracycline binds proximally to switch I and II in EF-Tu, illustrated by the superposition of EF-Tu in the GTP- and GDP-bound states onto the trypsin-modified EF-Tu•GDP•tetracycline X-ray structure. Switch I is highlighted in blue, switch II in green, and the P-loop in yellow. The structures aligned to domain I of the EF-Tu•GDP•tetracycline crystal structure (PDB ID 2HCJ) [21] were the *E*. *coli* homology model of the GTP-bound structure based on the crystal structure of *T*. *aquaticus* EF-Tu•GTP (PDB ID 1EFT) [34], and the EF-Tu•GDP crystal structure (PDB ID 1EFC) [35].

Apart from targeting the bacterial ribosome, a tetracycline-binding pocket has also been reported in EF-Tu, suggesting that tetracycline does indeed affect the function of EF-Tu directly [15, 19–24]. The structure of a 1:1 complex of trypsin-modified EF-Tu•GDP and tetracycline, solved using X-ray crystallography, supports a putative role of tetracycline in interfering with efficient nucleotide exchange *in vivo* [21]. Tetracycline is bound to the GTPase domain and interacts with several key functional residues within conserved motifs found in the GTPase and ATPase super families (Fig 1A). Briefly, tetracycline is coordinated through a magnesium ion, which is an essential co-factor for nucleotide binding in EF-Tu [25]. The following features of EF-Tu are involved in hydrogen bonding interactions with tetracycline: the α-phosphate of GDP, Thr25 (*Escherichia coli* numbering), and Asp80. Thr25 belongs to the conserved sequence of the phosphate-binding (P)-loop ([G/A]X_4_GK[S/T]). Asp80 is part of the conserved switch II trigger sequence (DX_2_G). The switch II trigger sequence and the P-loop are the most important contributors to GTP binding in all GTPases, and guanine nucleotide tri-phosphate specificity is due to the aspartate residue in the switch II trigger sequence [26]. Both of these motifs are conserved in many ATPases and GTPases [27]. In addition, a stacking interaction occurs between Pro82 and tetracycline. This proline residue is invariant in translational GTPases [28, 29]. Based on the location and amino acids that tetracycline interacts with in EF-Tu, it was predicted that nucleotide binding and GTP hydrolysis would be affected [21]. No steric clashes in the superposition of the EF-Tu•GDP•tetracycline complex and the EF-Tu•EF-Ts complex (Fig 1B) were observed [21]. However, given that the P-loop and magnesium ion are important features in EF-Ts-stimulated nucleotide dissociation, the ability of EF-Ts to stimulate GDP dissociation might be impeded (Fig 1C) [25, 30]. For example, in EF-Tu, nucleotide dissociation is initiated by the release of the phosphate end of the nucleotide [31]. Further, since the tetracycline-binding pocket is conserved in many GTPases and ATPases, additional essential proteins could be affected by tetracycline. It is estimated that 10–18% of all gene products are P-loop NTPases [32]. In turn, this would explain the observed discrepancy between the MIC and IC_50_ for *in vitro* translation assays.

**Fig 2:**
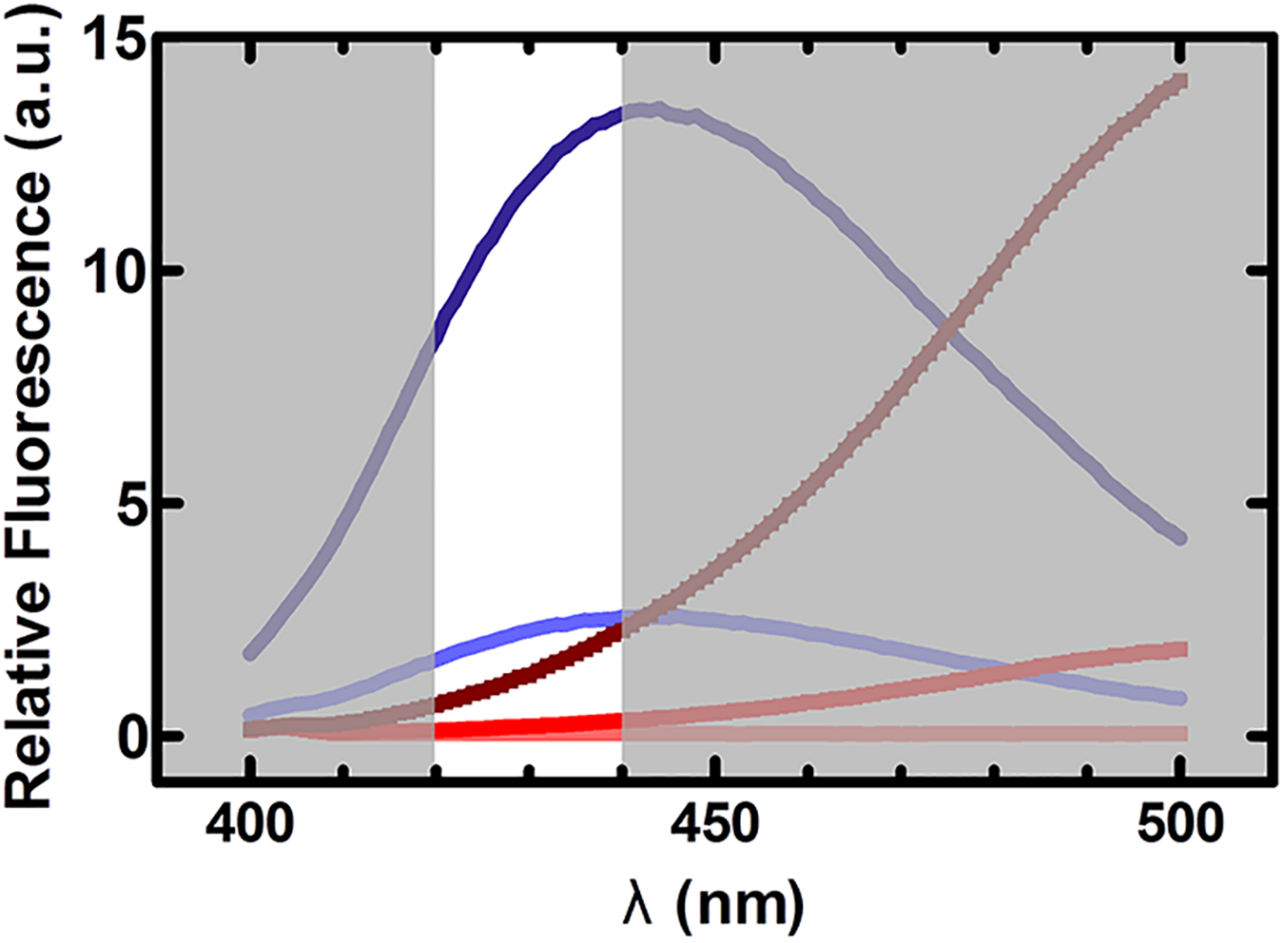
Fluorescence emission properties of tetracycline and mant-nucleotides. Comparison of the emission spectra of tetracycline and mant-GTP with the transmittance range of the 430 ± 10 nm band-pass filter (represented by the non-shaded area). Three concentrations of tetracycline, 0.1 μM (light pink), 10 μM (red), and 100 μM (brown) and two concentrations of mant-GTP, 1 μM (light blue) and 5 μM (dark blue), were excited at a wavelength of 335 nm.

Previously reported biochemical evidence suggests that tetracycline binding is able to modulate *E*. *coli* EF-Tu function [22, 23]. Using fluorescence spectroscopy, the ability of tetracycline to bind both *E*. *coli* EF-Tu and *Sulfolobus solfataricus* EF-1α was demonstrated [22]. This study also provided evidence that tetracycline binding might have an effect on nucleotide affinity as well as the rate of GTP hydrolysis in EF-1α. The effects of tetracycline on EF-1α were slight (a ~1.5-fold reduction in nucleotide affinity at 50 μM tetracycline and a ~25% decrease in the salt-stimulated GTPase activity at 120 μM tetracycline) but provided the basis for studying the effects of tetracycline on *E*. *coli* EF-Tu, which has a greater affinity for tetracycline than *S*. *solfataricus* EF-1α [22]. Due to the use of non-equilibrium methods (e.g. nitrocellulose filtration) and non-physiological conditions (e.g. salt-stimulated GTPase activity), the results reported for *S*. *solfataricus* EF-1α make it difficult to assess the effect of tetracycline on GTPase activity under *in vivo* conditions. Here, we adapted the fluorescence stopped-flow approach previously used [25, 30, 31, 36, 37] to study the kinetics of nucleotide binding in EF-Tu in order to gain detailed kinetic and thermodynamic information regarding the interaction of guanine nucleotides with EF-Tu in the presence of tetracycline. With the previously reported approach, it was not possible to observe the fluorescence of mant-labeled nucleotides in the presence of tetracycline due to the fluorescence properties of the antibiotic (Fig 2). Furthermore, using purified components from the *E*. *coli* translation machinery, we were able to determine the intrinsic and ribosome-stimulated GTPase activity of EF-Tu, as well as the stability of the ternary complex EF-Tu•GTP•Phe-tRNA^Phe^ in the presence of tetracycline, avoiding the use of non-physiological, high salt conditions.

To our knowledge, this is the first study that reports rate constants for the nucleotide-binding kinetics of EF-Tu in the presence of tetracycline. The results described here provide clear evidence that tetracycline does not affect translation through direct effects on the key enzymatic properties of EF-Tu, dismissing the observed interaction of tetracycline with EF-Tu as an exploitable target for antimicrobial drug development.

## Materials and methods

### Expression and purification of EF-Tu and EF-Ts

EF-Tu was expressed and purified to homogeneity as described in [36, 37]. All purification steps contained GDP to prevent EF-Ts co-purification. The concentration of EF-Tu was determined using the extinction coefficient of 32,900 M^-1^cm^-1^ at a wavelength of 280 nm. Protein purity was assessed by 12% SDS-PAGE stained with Coomassie Brilliant Blue.

EF-Ts was expressed as a fusion protein from the IMPACT I system (NE Biolabs), provided by Charlotte Knudsen (Åarhus, Denmark), as described in [37] and purified to homogeneity. The fusion protein contains a self-splicing intein and a chitin-binding domain that is removed during purification. The concentration and purity of EF-Ts were determined through 14% SDS-PAGE stained with Coomassie Brilliant Blue and ImageJ [38] was used to quantify the concentration through densitometry.

### Preparation of nucleotide-free EF-Tu

Nucleotide-free EF-Tu was prepared as described in [36, 37]. Briefly, EF-Tu•GDP was incubated with Buffer A (25 mM Tris-HCl, pH 7.5, 50 mM NH_4_Cl, and 10 mM EDTA) at 37^°^C for 30 min to promote the dissociation of GDP. Then, EF-Tu and GDP were separated on a Superdex 75 (GE healthcare) size exclusion column in Buffer B (25 mM Tris-HCl, pH 7.5, and 50 mM NH_4_Cl). Fractions containing EF-Tu were collected and the concentration was quantified spectroscopically (ε_280_ = 32,900 M^-1^cm^-1^). EF-Tu was diluted with Buffer C (50 mM Tris-HCl, pH 7.5, 70 mM NH_4_Cl, 30 mM KCl, and 7 mM MgCl_2_) prior to all experiments. All nucleotide-free EF-Tu was prepared the same day as the rapid-kinetics assays were performed.

### Rapid-kinetics measurements

A fluorescence stopped-flow apparatus (KinTek SF-2004) was used to determine rate constants as described in [25]. Buffer C was used for all stopped-flow measurements. Nucleotide binding to EF-Tu was determined through fluorescence resonance energy transfer from Trp184 (λ_ex_ = 280 nm) in EF-Tu to the mant-group on either mant-GTP or mant-GDP. The fluorescence signal was detected after passing through a 430 ± 10 nm band-pass filter (Edmund Optical). Nucleotide association rates were determined under pseudo first order conditions by titrating mant-GTP/mant-GDP against a constant concentration of nucleotide-free EF-Tu (~0.3 μM). The apparent rate constant for each mant-nucleotide concentration was determined by fitting the following one-exponential function to each fluorescence time course,

$$F=F_{\infty }+A\times \exp({-k}_{app}\times t)$$ (1)

where *F* is fluorescence at time *t*, *F*_*∞*_ is the final fluorescence, *A* is the amplitude and *k*_app_ is the apparent rate constant. The association rate constant was determined by plotting the apparent rate as a function of the nucleotide concentration. For all association and dissociation experiments, both syringes contained the respective concentration of tetracycline.

Dissociation rate constants were determined by forming the respective EF-Tu•mant-GTP/mant-GDP complex with 0.6 μM EF-Tu and 6 μM mant-nucleotide (syringe concentrations). Then, EF-Tu•mant-GTP/mant-GDP was chased with 60 μM of unlabeled nucleotide by rapidly mixing equal volumes of both solutions using the stopped-flow apparatus. The dissociation rate constant was determined by fitting each time course with a one-exponential function (Eq 1).

### GTPase assays

The rate of GTP hydrolysis was determined as in [39]. All GTPase assays were performed in Buffer C. Prior to measuring GTPase activity, [γ-^32^P]GTP (20 μM) was charged for 15 min at 37^°^C with phosphoenol pyruvate (3 mM), and pyruvate kinase (0.02 μg/μL). Then, EF-Tu (10 μM) alone or together with EF-Ts (0.02 μM or 0.2 μM) was added and the reaction was incubated for 15 min at 37^°^C and subsequently allowed to cool to room temperature for 5 min. EF-Ts was added into the reaction mixture to prevent GDP dissociation from being the rate-limiting step. The reaction was started by the addition of a solution containing the respective concentration of tetracycline with no ribosomes, 70S ribosomes (0.1 μM), or 50S ribosomes (0.1 μM). At each time point, a 5 μL aliquot of the reaction was quenched in 50 μL of perchloric acid (1 M) and dipotassium phosphate (2 mM). The liberated ^32^P_i_ was extracted using 400 μL isopropyl alcohol and 300 μL sodium molybdate (20 mM). The amount of hydrolyzed [γ-^32^P]GTP was determined by adding 200 μL of the organic phase to 2 mL of scintillation cocktail (EcoLite, MP Biomedical) in 10 mL scintillation vials and scintillation counting (Tri-Carb 2800TR Perkin Elmer). Background hydrolysis was determined and subtracted by using a reaction mixture that contained all components except EF-Tu.

### Hydrolysis protection assays

The stability of the EF-Tu•GTP•Phe-tRNA^Phe^ ternary complex was assessed as described in [36]. To this end, EF-Tu•GTP•Phe-tRNA^Phe^ ternary complex was formed in Buffer D (50 mM Tris-HCl, pH 7.5 (4^°^C), 70 mM NH_4_Cl, and 10 mM MgCl_2_) with EF-Tu (1.5 μM), [^14^C]Phe-tRNA^Phe^ (1.08 μM), GTP (1.5 mM), phosphoenol pyruvate (3 mM), and pyruvate kinase (0.17 μg/μL). [^14^C]Phe-tRNA^Phe^ was prepared as described in [36] by incubating tRNA^Phe^ (*E*. *coli* MRE 600, Sigma) with ATP (6 mM), inorganic pyrophosphatase (3 mM), phosphoenol pyruvate (3 mM), and pyruvate kinase (0.17 μg/μL) in Buffer E (25 mM Tris-Ac, pH 7.5 (room temperature), 11 mM Mg(OAc)_2_ 100 mM NH_4_OAc, 30 mM KOAc, and 1 mM dithiothreitol) for 30 min at 37^°^C. Then,[^14^C]-Phe (40 μM) and purified phenylalanyl-tRNA synthetase (~1 μM) were added to the solution and incubated for 20 min at 37^°^C. The reaction was quenched with the addition of 3 M KOAc (pH 4.5) to a final concentration of 0.3 M. Following phenol/chloroform extraction, the RNA was precipitated with 2.5 volumes of cold (-20^°^C) 100% ethanol overnight.

## Results and discussion

### The effect of tetracycline on the nucleotide-binding properties of EF-Tu

**Fig 3:**
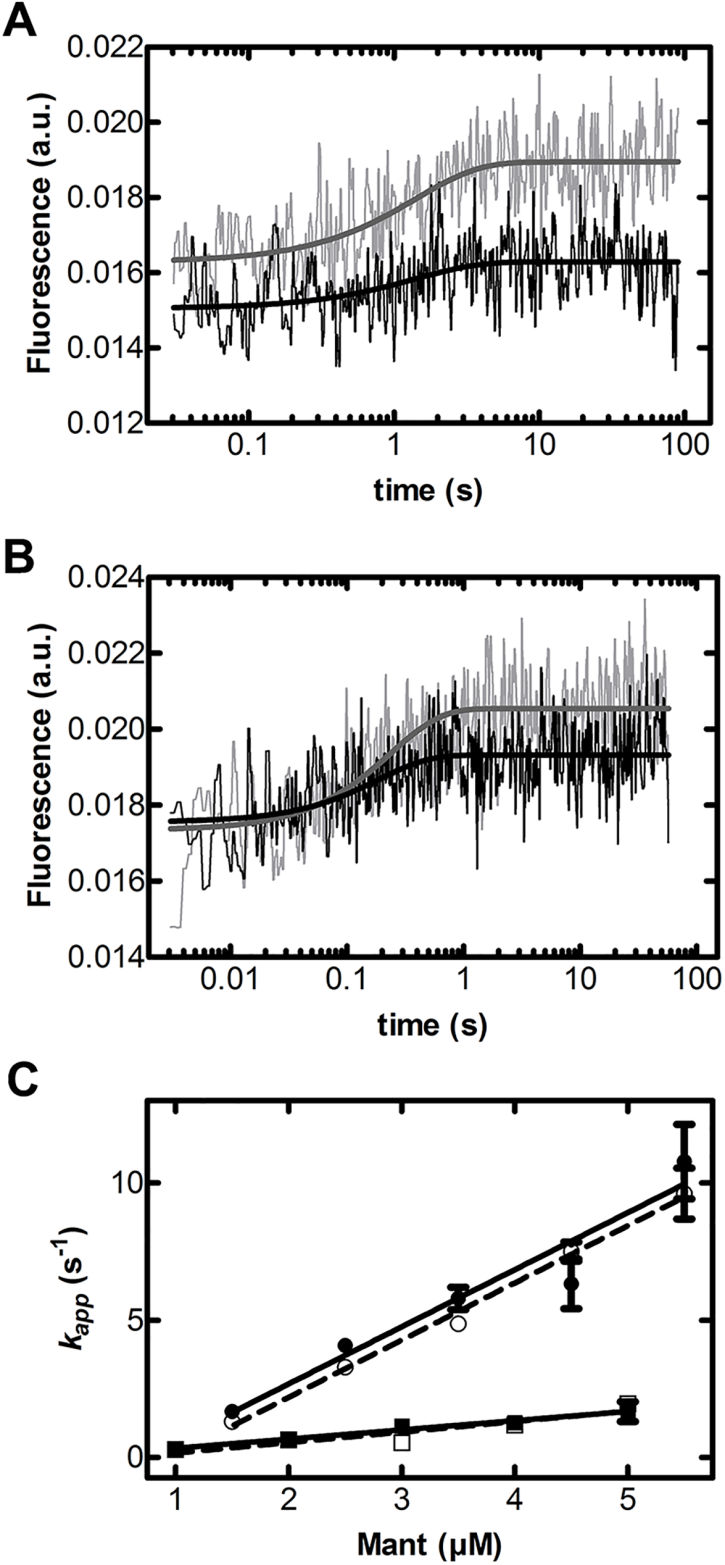
Effect of tetracycline on the association rate of EF-Tu and mant-nucleotides. Representative time courses of (A) mant-GTP (2 μM) or (B) mant-GDP (2.5 μM) binding to nucleotide-free EF-Tu (0.3 μM) in the presence of either 0 μM (grey) or 100 μM (black) tetracycline measured by exciting the single tryptophan residue at 280 nm and observing fluorescence resonance energy transfer to the mant group through a 430 ± 10 nm band-pass filter. (C) Concentration dependence of the apparent rate constant (*k*_app_) for mant-GTP (squares) or mant-GDP (circles) binding in the presence of either 0 μM tetracycline (open symbols, dashed line) or 100 μM tetracycline (solid symbols, solid line). Each *k*_app_ was determined by fitting individual time courses to a single exponential function, and the average of (n > 10) time courses determined at a given nucleotide concentration is plotted. Error bars represent standard error.

**Fig 4:**
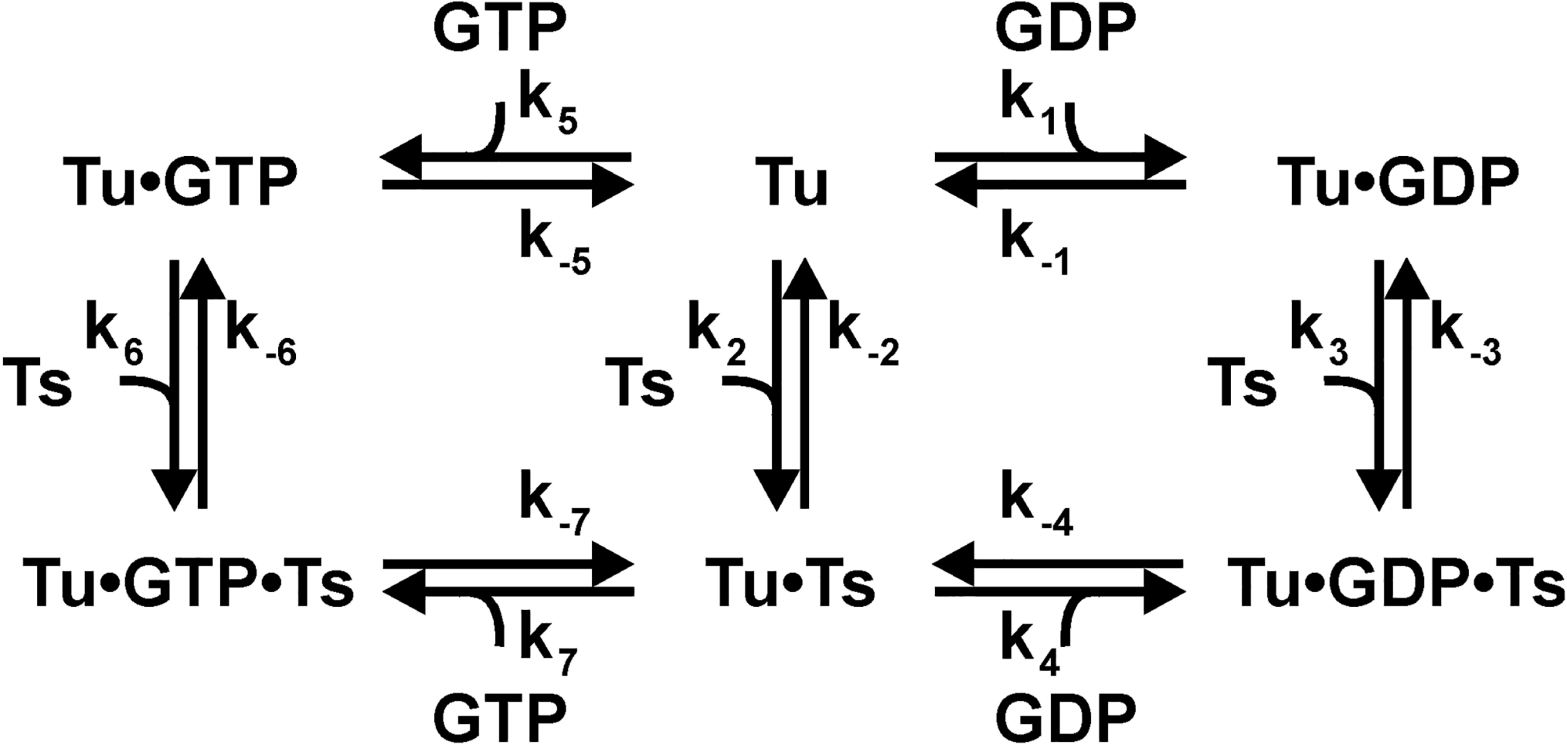
Kinetic mechanism of nucleotide exchange in EF-Tu.

The binding of tetracycline to the GTPase domain of EF-Tu (Fig 1A) was speculated to affect EF-Tu’s ability to bind and exchange guanine nucleotides [21], ultimately inhibiting efficient protein synthesis. Tetracycline was also reported to decrease the affinity of guanine nucleotides, suggested to be the result of tetracycline affecting the association rate constant of GTP/GDP [22]. Since tetracycline has a greater affinity for *E*. *coli* EF-Tu than *S*. *solfataricus* EF-1α, we predicted that the effect of tetracycline on nucleotide binding would be more pronounced in *E*. *coli* EF-Tu. We therefore modified the previously reported stopped-flow approach, described in [25, 30, 31, 36, 37], to enable the direct analysis of guanine nucleotide association and dissociation kinetics in EF-Tu. The major challenge to this approach is the autofluorescence of tetracycline which, at sufficiently high concentrations, overwhelms the mant fluorescence signal that reports binding of the respective nucleotide to EF-Tu [25]. A close inspection of the fluorescence spectra of mant-nucleotides and tetracycline when excited at 335 nm reveals that the majority of the tetracycline autofluorescence occurs at wavelengths greater than 450 nm, whereas the florescence maximum of mant lies at 440 nm (Fig 2). In the past, mant fluorescence was recorded using 400 nm long-pass cut-off filters. Based on these spectral properties, we used a 430 ± 10 nm band-pass filter which is optimal to reduce tetracycline emission and to still observe the fluorescence emission of the mant group (Fig 2) with high enough sensitivity to obtain time-resolved fluorescence changes of mant-GTP/mant-GDP (Fig 3). This modification of the well-established approach to dissect the nucleotide-binding kinetics in *E*. *coli* EF-Tu [25, 30, 31, 36, 37] allowed us to perform a detailed analysis of the association and dissociation kinetics of GTP and GDP, and of EF-Ts-stimulated nucleotide exchange in EF-Tu according to the kinetic scheme shown in Fig 4.

### Nucleotide association (*k*_1_ and *k*_5_)

**Fig 5:**
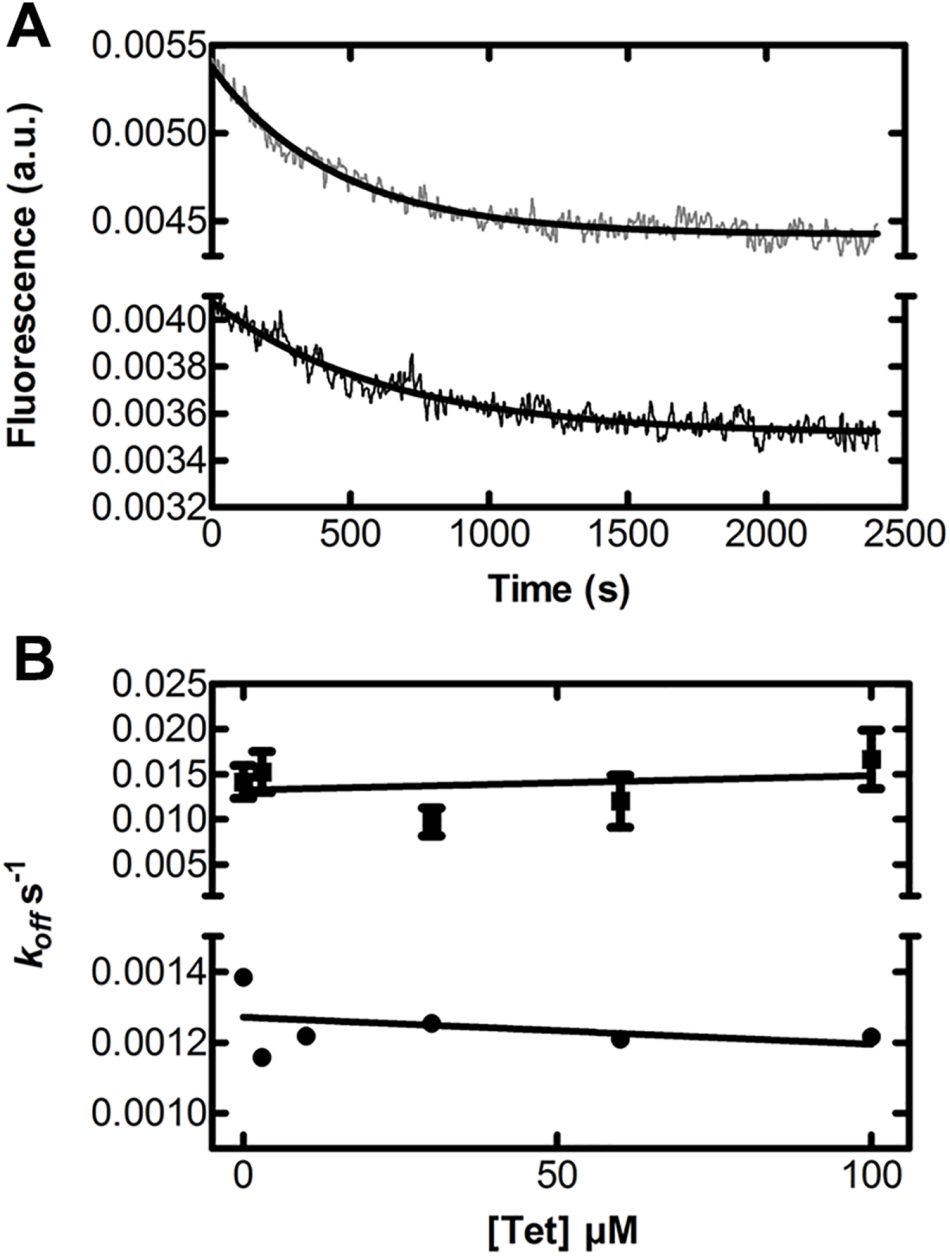
Effect of tetracycline on the dissociation rate of EF-Tu and mant-nucleotides. Panel (A) shows a representative time course of GDP dissociation from EF-Tu (0.3 μM) in the presence of either 0 μM tetracycline (grey) or 100 μM tetracycline (black). The concentration dependence of the *k*_-5_ (GTP, squares) or *k*_-1_ (GDP, circles) as a function of tetracycline concentration is shown in panel (B). Fluorescence resonance energy transfer was monitored by exciting the single tryptophan residue present in EF-Tu and monitoring mant fluorescence through a 430 ± 10 nm band-pass filter. Each rate constant is an average of (n > 9) time courses fit to a single exponential function, and error bars are standard errors.

The association rate constants for GDP (*k*_1_) and GTP (*k*_5_) were determined by titrating mant-guanine nucleotides against a constant concentration of nucleotide-free EF-Tu. At each nucleotide concentration, the apparent association rate constant (*k*_app_) was determined in the presence and absence of 100 μM tetracycline (Fig 5C). By plotting the apparent rate constants with respect to the nucleotide concentration, the respective rate constant was determined (Table 1). The GTP association rate constant in the absence of tetracycline was *k*_5_ = 3.9 ± 0.1 × 10^5^ M^-1^s^-1^. In the presence of 100 μM tetracycline, no change was observed (*k*_5, tet_ = 3.4 ± 0.1 × 10^5^ M^-1^s^-1^). Both of these GTP association rate constants are in agreement with the previously reported value of *k*_5_ = 5 ± 1 × 10^5^ M^-1^s^-1^ [25]. Similarly, the GDP association rate constant *k*_1_ is not affected by the addition of 100 μM tetracycline (*k*_1_ = 2.1 ± 0.1 × 10^6^ M^-1^s^-1^ and *k*_1_,_tet_ = 2.1 ± 0.3 × 10^6^ M^-1^s^-1^), and both rate constants are consistent with the earlier reported value of *k*_1_ = 2.0 ± 0.5 × 10^6^ M^-1^s^-1^ [25]. Therefore, tetracycline has no effect on the association rate constants of either GTP or GDP to *E*. *coli* EF-Tu and, in turn, on the thermodynamics of this interaction.

**Table 1:** Kinetic parameters of nucleotide binding in EF-Tu in the presence of 0 μM and 100 μM tetracycline.

Rate constant	0 μM tetracycline	100 μM tetracycline
*k*_1_ (× 10^6^ M^-1^s^-1^)	2.1 ± 0.1	2.1 ± 0.3
*k*_5_ (× 10^5^ M^-1^s^-1^)	3.9 ± 0.1	3.4 ± 0.1
*k*_-1_ (× 10^−3^ s^-1^)	1.4 ± 0.1	1.2 ± 0.1
*k*_-5_ (× 10^−2^ s^-1^)	1.4 ± 0.2	1.7 ± 0.3
*k*_3_*/*(1 + *k*_-3_*/k*_-4_) (× 10^6^ M^-1^s^-1^)	13.4 ± 1.1	13.9 ± 0.5
*k*_6_*/*(1 + *k*_-6_*/k*_-7_) (× 10^6^ M^-1^s^-1^)	21.9 ± 1.4	19.8 ± 1.1

### Nucleotide dissociation (*k*_-1_ and *k*_-5_)

The dissociation rate constants for GDP (*k*_-1_) and GTP (*k*_-5_) were measured by chasing EF-Tu•mant-GTP/mant-GDP with excess unlabeled GTP/GDP [25]. Under these conditions, dissociation of the mant-labeled nucleotide is rate limiting and the binding of unlabeled nucleotide is rapid, effectively preventing rebinding of the labeled nucleotide. Therefore, the observed dissociation rate is the rate constant of this first-order dissociation reaction (*k*_-1_, *k*_-5_). Fig 5A shows the obtained dissociation time course of GDP in the absence of tetracycline. When carried out in the presence of increasing concentrations of tetracycline, no change of the rate constant for either GTP (*k*_-5_ *=* 1.7 ± 0.3 × 10^−2^ s^-1^) or GDP (*k*_-1_ = 1.2 ± 0.1 × 10^−3^ s^-1^) was observed (Fig 5B). The obtained dissociation rate constants (summarized in Table 1) agree with the previously reported rate constants of *k*_-5_ = 3 ± 1 × 10^−2^ s^-1^ for GTP dissociation and *k*_-1_ = 2 ± 1 × 10^−3^ s^-1^ for GDP dissociation [25]. These results demonstrate that tetracycline does not interfere with the spontaneous dissociation of guanine nucleotides from EF-Tu (*k*_-1_ and *k*_-5_).

### EF-Ts stimulated nucleotide dissociation from EF-Tu (*k*_3_/(1 + *k*_-3_/*k*_-4_) and *k*_6_/(1 + *k*_-6_/*k*_-7_))

**Fig 6:**
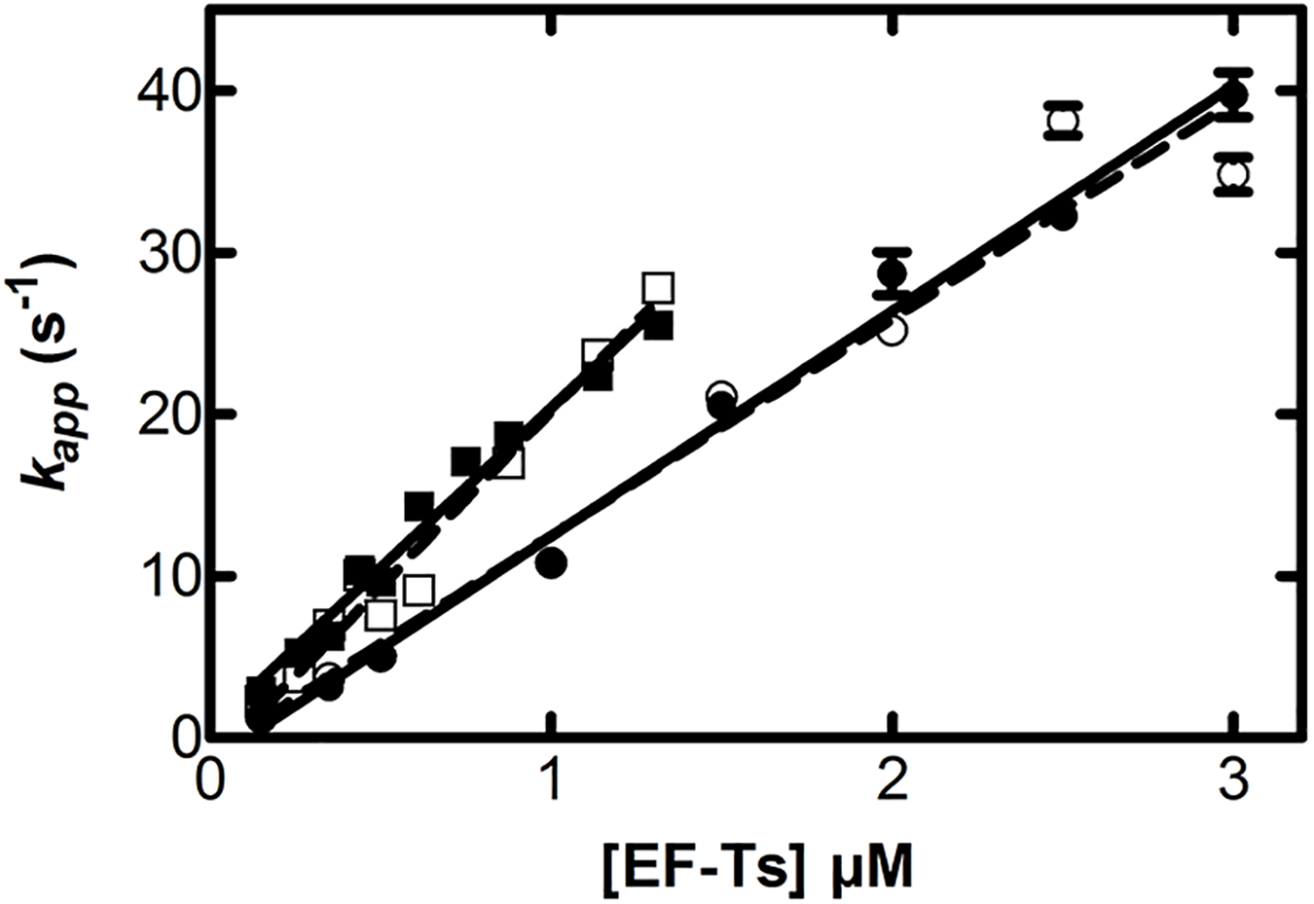
Effect of tetracycline on EF-Ts-stimulated dissociation of mant-nucleotides from EF-Tu. The EF-Ts dependence of the apparent dissociation rates (*k*_app_) for mant-GTP (squares) and mant-GDP (circles) dissociation from EF-Tu (0.15 μM) is shown in the presence (solid symbols, solid line) and absence (open symbols, dashed line) of 100 μM tetracycline. Fluorescence resonance energy transfer from the mant group to the single tryptophan in EF-Tu was observed through a 430 ± 10 nm band-pass filter by exciting the single tryptophan in EF-Tu at 280 nm. Each *k*_app_ is the average (n > 11) of time courses at that EF-Ts concentration. The error bars indicate the standard error.

Under *in vivo* conditions, the exchange of nucleotides in EF-Tu requires an additional translation factor, EF-Ts. The action of nucleotide exchange factor EF-Ts is required because spontaneous dissociation of nucleotides, in particular GDP, from EF-Tu is too slow to sustain *in vivo* protein synthesis rates. To investigate a potential effect of tetracycline on this physiologically relevant step, we performed an EF-Ts titration of the stimulated nucleotide exchange reaction. We determined the apparent rate of nucleotide dissociation from EF-Tu at increasing concentrations of EF-Ts in the presence of a constant amount of EF-Tu•mant-GTP/mant-GDP, similar to the approach describe above [25]. Consistent with the coupled equilibria in Fig 4, we observed a linear initial phase (Fig 6) under low concentrations of EF-Ts, which represents the combined rate constants for the formation of the EF-Tu•GTP/GDP•EF-Ts complex and the dissociation of the nucleotide (Fig 4). This approach allows us to assay if any of the EF-Ts related steps are affected by tetracycline, as a change in the rate constant of either of the contributing steps will alter the slope of the concentration dependence. Interestingly, the slope of the combined rate constants in the presence and absence of 100 μM tetracycline is unchanged (summarized in Table 1). The value of the combined rate constants for stimulated GTP dissociation was *k*_6_*/*(1 + *k*_-6_*/k*_-7_) = 21.9 ± 1.4 × 10^6^ M^-1^s^-1^ in the absence of tetracycline and *k*_6_*/*(1 + *k*_-6_*/k*_-7_) = 19.8 ± 1.1 × 10^6^ M^-1^s^-1^ in the presence of 100 μM tetracycline. Both of these values agree with earlier reported work (*k*_6_*/*(1 + *k*_-6_*/k*_-7_)_tet_ = 20 × 10^6^ M^-1^s^-1^) and indicate that tetracycline does not affect EF-Ts-stimulated dissociation of GTP from EF-Tu [25]. Similarly, the combined rate constants for the stimulated dissociation of GDP were determined to be *k*_3_*/*(1 + *k*_-3_*/k*_-4_) = 13.4 ± 1.1 × 10^6^ M^-1^s^-1^ in the absence of tetracycline and *k*_3_*/*(1 + *k*_-3_*/k*_-4_)_tet_ = 13.9 ± 0.5 × 10^6^ M^-1^s^-1^ in the presence of 100 μM tetracycline, which is in excellent agreement with the reported value of *k*_3_*/*(1 + *k*_-3_*/k*_-4_) = 16 × 10^6^ M^-1^s^-1^ [25]. Therefore, our results indicate that tetracycline does not affect either the interaction of EF-Tu and EF-Ts (*k*_-3_ and *k*_-6_) or the subsequent nucleotide release steps from the EF-Tu•GTP/GDP•EF-Ts ternary complex (*k*_4_, *k*_-4_ and *k*_7_, *k*_-7_).

In summary, the detailed kinetic analysis of the nucleotide-binding properties of EF-Tu in the presence of up to 100 μM tetracycline support the notion that this essential step in the functional cycle of EF-Tu is not affected by tetracycline. The tetracycline concentration used here is much greater than the peak plasma concentration of a single standard administered dose of tetracycline in humans, which is 1.02 μg/mL (2.29 μM) [40]. Therefore, we feel confident that tetracycline does not target nucleotide binding in EF-Tu as part of its mode of action. Our results are in contrast to data reported by Lamberti et al. [22], which reported a slight (1.5- to 1.7-fold) decreased affinity in EF-1α for guanine nucleotides in the presence of 50 μM tetracycline. However, non-equilibrium methods were used by Lamberti et al. and the effect on the association rate constant was not directly measured [22]. Furthermore, our observations are supported by a computational study suggesting that tetracycline binding to EF-Tu causes only a small change in free energy and is facilitated indirectly via the magnesium ion and GDP [19].

### Tetracycline has no effect on the GTPase activity of EF-Tu

Although the nucleotide-binding properties of EF-Tu are not altered in the presence of tetracycline, the fact that tetracycline binds to the G domain of EF-Tu and interacts with the P-loop and the switch II trigger sequence (Fig 1C) gives rise to the hypothesis that its mode of action might include a direct effect on GTP hydrolysis by EF-Tu [21, 41]. Consistent with such a role, the previously reported salt-stimulated GTPase activity of *S*. *solfataricus* EF-1α was reduced by ~25% in the presence of 120 μM tetracycline [22]. However, decreasing the already extremely slow intrinsic GTP-hydrolysis rate of EF-Tu seems an unlikely additional mode of tetracycline antibiotic action. To investigate this further, we used physiologically relevant buffer conditions to determine if tetracycline had an effect, not only on the intrinsic, but also on the 70S ribosome and 50S ribosomal subunit-stimulated GTPase activity of EF-Tu.

### Intrinsic GTPase activity

**Fig 7:**
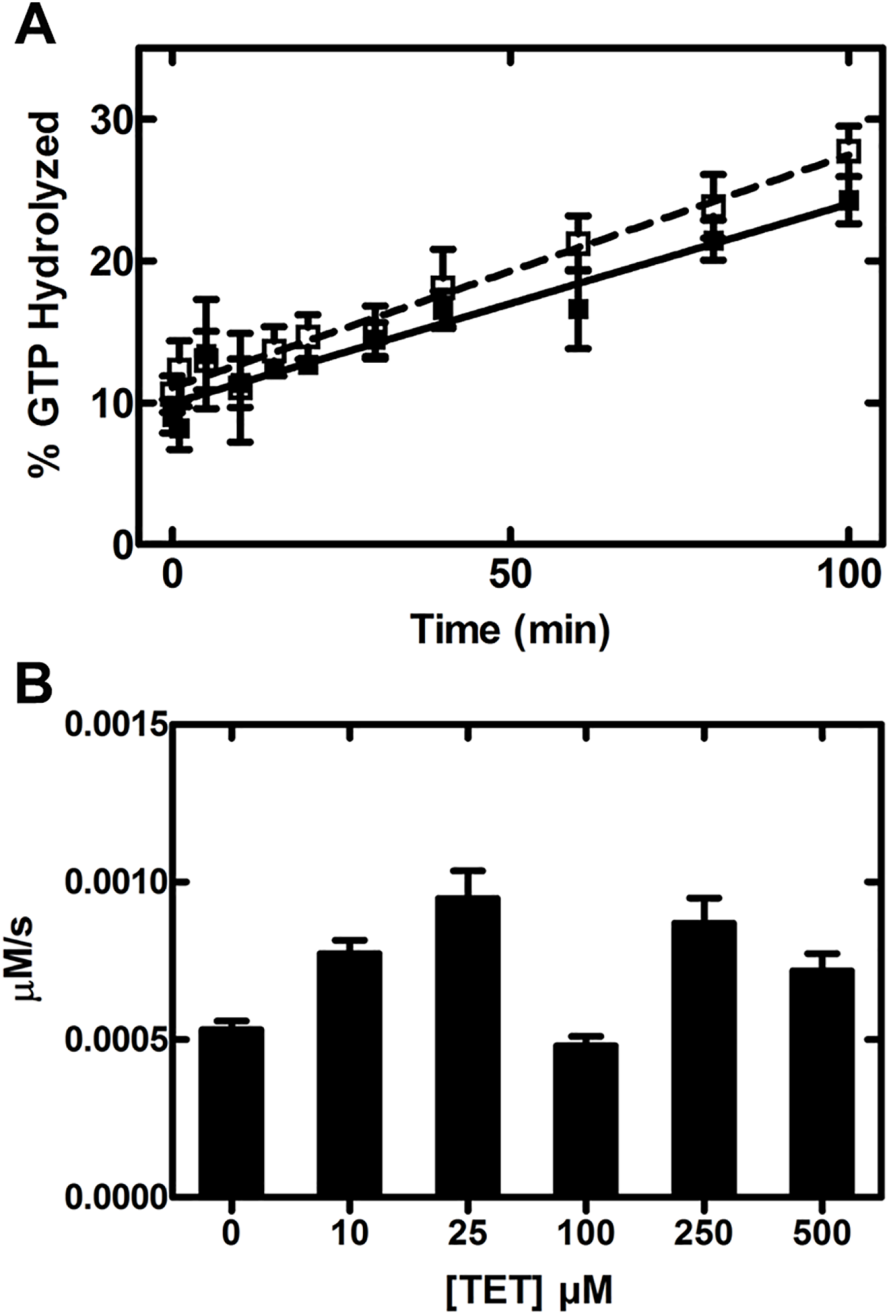
Effect of tetracycline on the intrinsic GTPase activity. (A) The linear phase of multiple turnover GTP hydrolysis reactions using 10 μM EF-Tu and 0.2 μM EF-Ts are shown for 0 μM (open squares, dashed line) and 100 μM (solid squares, solid line) tetracycline. (B) Comparison of the multiple turnover GTP hydrolysis rates at different tetracycline concentrations. Data shown are averages (n = 3) and the error bars indicate the standard error.

To investigate if tetracycline affects intrinsic GTP hydrolysis by EF-Tu, we determined the multiple turnover GTPase activity of EF-Tu at increasing concentrations (up to 500 μM) of tetracycline. Rates of multiple turnover GTP hydrolysis (*k*_GTPase_) were determined from the initial linear phase of the time course, both in the presence and absence of tetracycline (Fig 7A, Table 2). Our results in the absence of tetracycline are consistent with previous work using the same buffer [39]. In addition, increasing the tetracycline concentration up to 500 μM (Fig 7B) did not reduce the observed multiple turnover hydrolysis rate of intrinsic GTP hydrolysis (summarized in Table 2). This further supports our observation that tetracycline at concentrations higher than 100 μM is not interfering with the EF-Ts-mediated nucleotide exchange reaction. Furthermore, our results presented here demonstrate that tetracycline, although able to bind in the vicinity of the γ-Phosphate of the bound GTP, does not alter the intrinsic GTPase activity of EF-Tu.

**Table 2:** Effect of 100 μM tetracycline on the intrinsic, 70S-, and 50S-stimulated GTPase activity of EF-Tu.

Multiple turnover rate	0 μM tetracycline	100 μM tetracycline
*k*_GTPase_ (× 10^−4^ μM/s)	7.67 ± 1.33	6.67 ± 1.33
*k*_GTPase, 0.02μM EF-Ts_ (× 10^−4^ μM/s)	5.33 ± 0.67	4.67 ± 0.67
*k*_GTPase, 0.2μM EF-Ts_ (× 10^−4^ μM/s)	5.33 ± 0.33	4.67 ± 0.33
*k*_GTPase,70S_ (× 10^−3^ μM/s)	2.67 ± 0.20	2.17 ± 0.23
*k*_GTPase,50S_ (× 10^−3^ μM/s)	1.0 ± 0.3	1.3 ± 0.3

### Ribosome-stimulated GTPase activity

**Fig 8:**
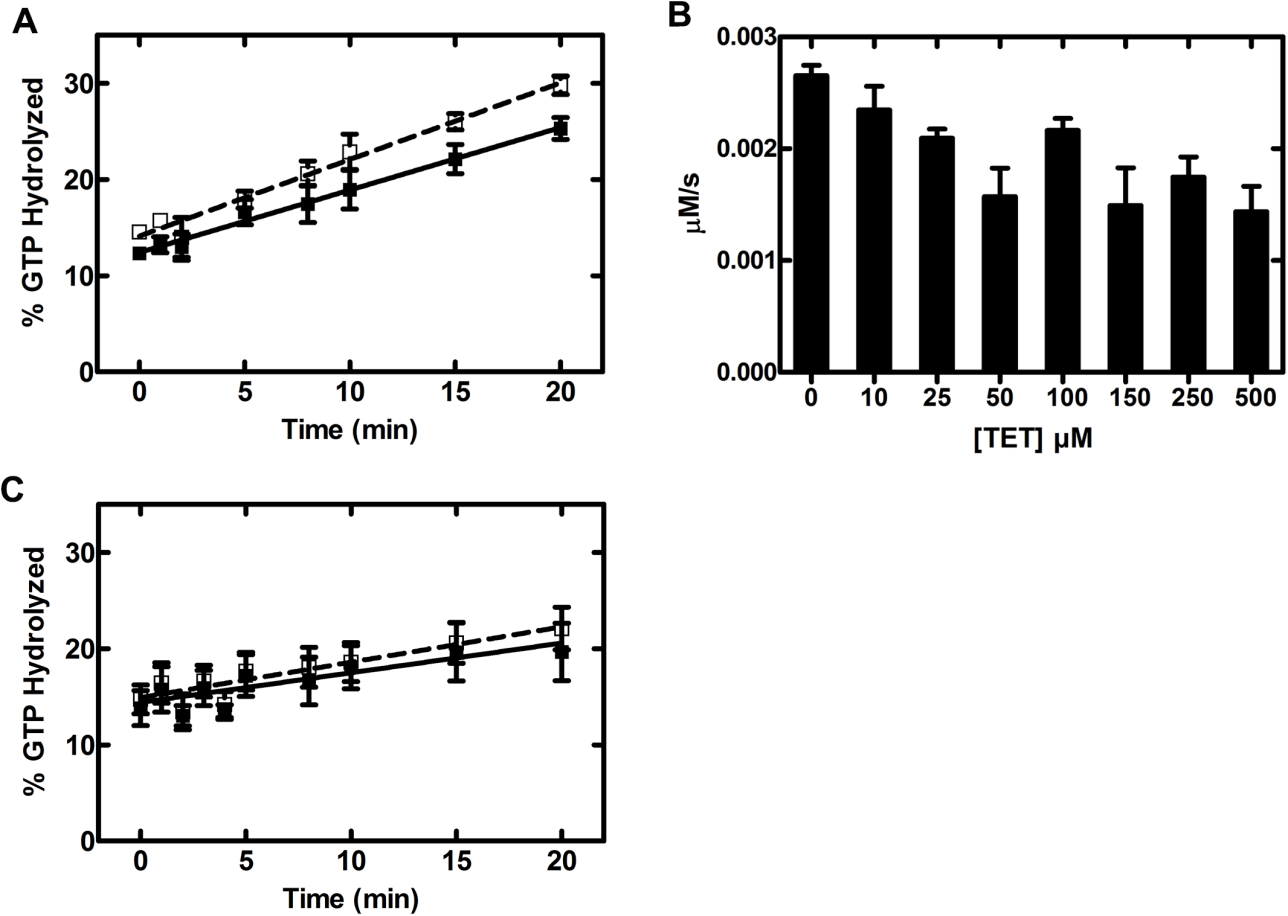
Ribosome-stimulated GTPase activity. Effect of tetracycline on the 70S (0.1 μM) and 50S (0.1 μM) -stimulated GTPase activity of EF-Tu (10 μM) in the presence of EF-Ts (0.02 μM). Panel (A) is the linear phase of the time course of multiple turnover GTP hydrolysis of EF-Tu stimulated by the 70S ribosome in the presence (100 μM, solid squares, solid line) and absence (open squares, dashed line) of tetracycline. Panel (B) shows the dependence of the rate of 70S-stimulated GTP hydrolysis on the concentration of tetracycline. The linear phase of 50S stimulated GTP hydrolysis reaction is shown in panel (C) in the presence (100 μM, solid squares, solid line) and absence (open squares, dashed line) of tetracycline. Each point on the plots is the average of (n = 3) independent experiments and the error bars represent the standard error.

The multiple turnover GTPase activity of EF-Tu can be stimulated ~2-fold by the presence of empty 70S ribosomes [23, 39], providing a sensitive measure for tetracycline interfering with the interaction of EF-Tu•GTP. Similar to the reported intrinsic GTPase activity, rates of hydrolysis were determined from the initial phase of the respective GTP hydrolysis time courses. The 70S-stimulated multiple turnover GTP hydrolysis activity of EF-Tu was *k*_GTPase,70S_ = 2.67 ± 0.20 × 10^−3^ μM/s (Fig 8A and Table 2). In the presence of increasing concentrations of tetracycline, up to 500 μM (Fig 8B), the multiple turnover GTP hydrolysis activity remains essentially unaffected. With a *k*_70S, tet_ at 100 μM of 2.17 ± 0.23 × 10^−3^ μM/s, the rate of 70S-stimulated GTPase activity in EF-Tu is similar under all tetracycline conditions tested. Consistent with this, the 50S-stimulated GTP-hydrolysis activity of EF-Tu is unaffected by 100 μM tetracycline (*k*_50s_ = 1.0 ± 0.3 × 10^−3^ μM/s and *k*_50S,tet_ = 1.3 ± 0.3 × 10^−3^ μM/s, summarized in Fig 8C and Table 2). This is not surprising, as the 70S-stimulated GTPase activity of EF-Tu is mainly due to the interaction of EF-Tu with the GTPase Activating Center (GAC) and the sarcin-ricin loop (SRL), including ribosomal proteins L7/L12 located on the 50S [42]. Our observation that the 70S ribosome, which is the cellular target of EF-Tu, is able to stimulate the GTPase activity of EF-Tu, even at 500 μM, is in contrast to Lamberti et al. [22] who reported an effect of tetracycline on the ribosome-independent salt-stimulated GTPase activity of EF-1α. The observed 25% reduction might be specific to their use of 3.6 M NaCl to stimulate the GTPase activity of *S*. *solfataricus* EF-1α. Therefore, the salt-stimulated GTPase activity could involve an alternative mechanism that might be sensitive to the presence of tetracycline but that is not relevant under physiological conditions.

### Tetracycline does not alter EF-Tu•GTP•Phe-tRNA^Phe^ stability

**Fig 9:**
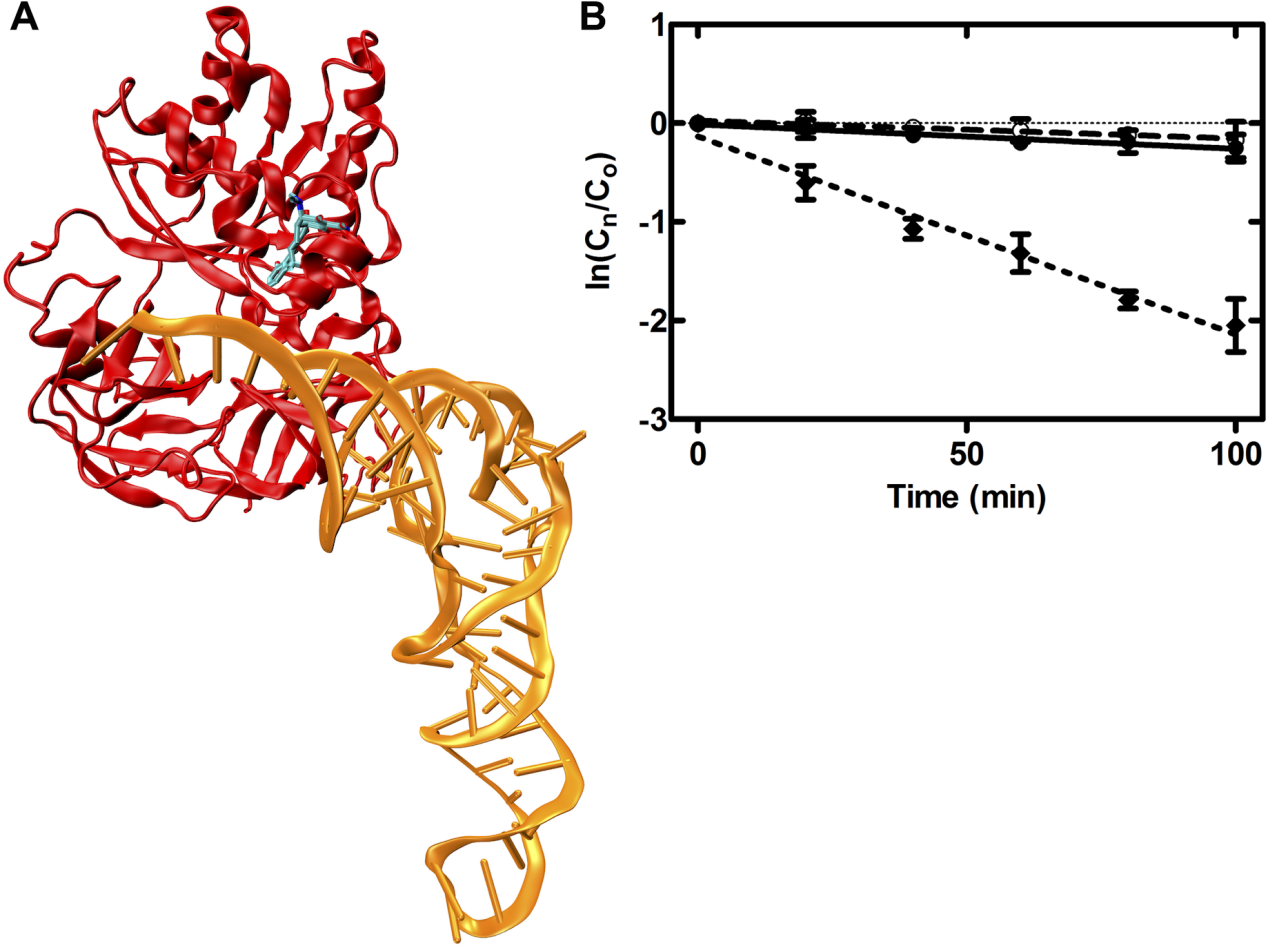
Effect of tetracycline on EF-Tu•GTP•Phe-tRNA^Phe^ stability. (A) Model of the EF-Tu•GTP•Phe-tRNA^Phe^ complex interacting with tetracycline (coloured by atom). EF-Tu is shown in red and Phe-tRNA^Phe^ is orange. The crystal structure of the EF-Tu•GTP•aa-tRNA complex (PDB ID 1OB2) was superimposed onto domain I of the EF-Tu•GDP•tetracycline complex (PDB ID 2HCJ) [21]. (B) Time dependence of the spontaneous hydrolysis of the amino-ester bond obtained by incubating Phe-tRNA^Phe^ (1.08 μM) at 37°C with no EF-Tu (solid diamonds, dotted line), with EF-Tu•GTP (1.5 μM) and no tetracycline (open circles, dashed line), or EF-Tu•GTP (1.5 μM) and 100 μM tetracycline (solid circles, solid line).

In the active GTP bound state, EF-Tu has a high affinity for aminoacyl(aa)-tRNA (K_D_ ≈ 10^−8^ M) and forms the EF-Tu•GTP•aa-tRNA ternary complex [43]. The interaction between aa-tRNA and EF-Tu involves binding of the aminoacylated 3’-end of the tRNA into the cleft between domain I and II. The 5’-end of the tRNA body is bound by the junctions of the three domains of EF-Tu [44]. While the aa-tRNA is bound by EF-Tu as part of the ternary complex (Fig 9A), the aminoacyl-ester bond between the amino acid and the tRNA body is protected from spontaneous hydrolysis (~10-fold) [36]. This effect is highly sensitive to structural perturbation of the amino acid binding pocket as well as slight changes in the on- and off-rates of the aa-tRNA. The tetracycline binding site is proximate to switch I and II, which undergo structural rearrangements upon GTP binding to EF-Tu and form part of the aa-tRNA interaction surface of EF-Tu. Therefore, we wanted to investigate if the binding of tetracycline to EF-Tu might perturb the aa-tRNA interaction. To this end, we monitored the stability of the aminoacyl-ester bond of Phe-tRNA^Phe^ as previously described [36] in the presence of increasing tetracycline concentrations (Fig 9B). As reported by De Laurentiis et al. [36], the half-life of the [^14^C]Phe-tRNA^Phe^ was ten-fold greater in the presence of EF-Tu•GTP than in the absence of EF-Tu. Increasing the tetracycline concentration to 100 μM had no effect on EF-Tu’s ability to protect the highly sensitive aminoacyl-ester bond against spontaneous hydrolysis (Fig 9B and Table 3). This is supported by the observation that tetracycline does not impede the delivery of the EF-Tu•GTP•aa-tRNA ternary complex to the ribosome [15]. This observation suggests that either tetracycline binds to *E*. *coli* EF-Tu and does not affect aa-tRNA binding or that tetracycline does not have a high enough affinity to the ternary complex under physiologically relevant conditions.

**Table 3:** Effect of tetracycline on EF-Tu mediated protection of the Phe-tRNA^Phe^ against spontaneous hydrolysis.

Reaction conditions	Half-life (min)
0 μM EF-tu, 0 μM Tet	35 ± 4
1.5 μM EF-Tu, 0 μM Tet	382 ± 64
1.5 μM EF-Tu, 3 μM Tet	542 ± 46
1.5 μM EF-Tu, 30 μM Tet	483 ± 45
1.5 μM EF-Tu, 100 μM Tet	282 ± 72

## Conclusion

Our data reported here demonstrates that tetracycline does not affect the nucleotide-binding properties of EF-Tu, nor the ability of EF-Ts to stimulate nucleotide dissociation. Furthermore, no effect on the intrinsic, 70S- and 50S-stimulated multiple turnover GTP hydrolysis activity of EF-Tu could be detected. Similarly, the formation or stability of the EF-Tu•GTP•aa-tRNA ternary complex is insensitive to the presence of tetracycline under physiologically relevant conditions. These findings suggest that during therapeutic use of tetracycline, EF-Tu is not a direct target of the antibiotic because the observed peak plasma concentration of tetracycline is more than 100-times lower than the concentrations used in our work [40]. Our observations also indicate that although tetracycline is able to bind to the trypsin-modified fragment of EF-Tu, it does not influence the functional cycle of EF-Tu. As a consequence, direct tetracycline binding to EF-Tu as an exploitable target for antimicrobial drugs likely has to be dismissed.

Interestingly, the reported binding pocket of tetracycline is conserved in many other GTPases and ATPases. However, the predicted main contributor to tetracycline binding in EF-Tu is the conserved, nucleotide-bound magnesium ion [19]. If tetracycline binds other GTPases and ATPases through a divalent metal ion interaction in a similar binding pocket as in EF-Tu, our data suggest that tetracycline will likely also not affect their function. Therefore, the observed discrepancy between the MIC and IC_50_ for tetracycline must be caused by other tetracycline binding sites on the 30S and 50S ribosomal subunits, likely through perturbation of different processes such as ribosome biogenesis or translation termination [17, 45]. Further detailed mechanistic studies regarding these secondary-binding sites will be necessary and could provide novel structural and mechanistic targets for the development of new classes of antibacterial compounds.

## Supporting information

S1 File Effect of tetracycline on the association rate of EF-Tu and GTP/GDP. This file contains the processed data used to calculate the means and standard errors shown in Fig 3C.
(XLSX)

S2 File GTP and GDP dissociation from EF-Tu under different tetracycline concentrations. This file contains the processed data used to calculate the means and standard errors shown in Fig 5B.
(XLSX)

S3 File EF-Ts stimulated dissociation of GTP and GDP from EF-Tu with and without tetracycline. This file contains the means and standard errors displayed in Fig 6, as well as the raw fluorescence data used to calculate those values.
(XLSX)

S4 File Effect of tetracycline on intrinsic GTPase activity of EF-Tu. This file contains the processed data used to calculate the means and standard errors shown in Fig 7A and 7B.
(XLSX)

S5 File Ribosome-stimulated GTPase activity of EF-Tu. This file contains the processed data used to calculate the means and standard errors shown in Fig 8A, 8B and 8C.
(XLSX)

S6 File Effect of tetracycline on EF-Tu•GTP•Phe-tRNA^Phe^ stability. This file contains the processed data used to calculate the means and standard errors shown in Fig 9B.
(XLSX)
